# Assessment of Left Ventricular Function After Percutaneous Coronary Intervention for Chronic Total Occlusion

**DOI:** 10.1016/j.jscai.2024.102460

**Published:** 2025-01-21

**Authors:** Yasser M. Sammour, Rody G. Bou Chaaya, Chloe Kharsa, Jerrin Philip, Taha Hatab, Sahar Samimi, Joseph Elias, Momin Islam, Gal Sella, Joe Aoun, Sachin S. Goel, Neal S. Kleiman, Alpesh R. Shah

**Affiliations:** Department of Cardiology, Houston Methodist DeBakey Heart and Vascular Center, Houston, Texas

**Keywords:** chronic total occlusion, left anterior descending coronary artery, left circumflex artery, left ventricular ejection fraction, left ventricular function, percutaneous coronary intervention, right coronary artery

## Abstract

**Background:**

The impact of percutaneous coronary intervention (PCI) for chronic total occlusion (CTO) on left ventricular ejection fraction (LVEF) remains controversial.

**Methods:**

We included patients who underwent CTO PCI (2018-2022) with reported baseline and follow-up LVEF (window 1-18 months). Stratified analyses according to procedural success, baseline LVEF, and target vessel were performed. Logistic regression analysis was performed to assess predictors of LVEF improvement.

**Results:**

We included 142 patients with available LVEF data, of whom 121 had successful CTO PCI (85.2%). Overall, mean age was 65.4 ± 10.3 years, 76.1% were men, and 81.0% were White. The attempted CTO vessel was left anterior descending in 31.7%, left circumflex in 17.6%, and right coronary artery in 50.0% of patients. The median time from PCI to follow-up echocardiogram was 8.4 months (IQR, 4.4-12.4). After successful CTO PCI, mean LVEF increased from a baseline of 48.2% ± 15.4% to 51.8% ± 14.2% (ΔLVEF 3.6%; *P* < .001). Among patients with depressed baseline LVEF <50%, there was greater improvement in LVEF from 32.6% ± 9.7% to 40.0% ± 12.9% (ΔLVEF 7.6%; *P* < .001), including 48.0% with ≥10% improvement. There was no change in LVEF after unsuccessful CTO PCI (54.6% ± 10.6% vs 55.2% ± 8.6%; *P* = .746). The ΔLVEF after successful CTO PCI to the left anterior descending, left circumflex, and right coronary artery was 2.6%, 4.0%, and 4.4%, respectively, overall, and 9.4%, 6.3%, 7.3% in patients with depressed baseline LVEF. Reduced baseline LVEF <50% was a strong independent predictor of LVEF improvement after successful CTO PCI (adjusted odds ratio, 5.60; 95% CI, 2.27-13.84; *P* < .001).

**Conclusions:**

Successful CTO PCI seems to be associated with modest LVEF improvement, which is more pronounced in patients with reduced baseline LVEF.

## Introduction

The impact of percutaneous coronary intervention (PCI) for chronically occluded coronary arteries on left ventricular (LV) function remains debated. Both the Evaluating Xience and Left Ventricular Function in Percutaneous Coronary Intervention on Occlusions After ST-Elevation Myocardial Infarction (EXPLORE) and Recovery of Left Ventricular Function After Stent Implantation in Chronic Total Occlusion of Coronary Arteries (REVASC) trials found no significant improvement in left ventricular ejection fraction (LVEF), LV end-diastolic volume, or segmental wall thickening with additional PCI for chronic total occlusion (CTO) lesions.^1^^,^^2^ Some studies suggest that revascularization may improve segmental wall thickening in the CTO territory.^3^^,^^4^ Additionally, observational studies and meta-analyses have suggested that successful CTO PCI may improve LV function, especially in patients with LVEF below 50%, potentially reversing adverse LV remodeling.^5^^,^^6^ Given the limited data, we sought to investigate the impact of CTO PCI on LVEF, and to identify which patients might benefit the most from these interventions.

## Methods

### Study patients

We included consecutive patients who underwent CTO PCI at the Houston Methodist DeBakey Heart and Vascular Center from January 2018 through December 2022 and who had baseline and follow-up LVEF measured within a window of 1 to 18 months after PCI. We excluded patients who had missing LVEF or reported LVEF outside that timeframe. This study was carried out in accordance with the appropriate ethical guidelines. The study was approved by the institutional review board at our hospital. Informed consent was waived due to the retrospective nature of our study.

### Case review and adjudication process

We constructed a hierarchical review and adjudication process. CTO was defined as total occlusion of a coronary artery and Thrombolysis in Myocardial Infarction (TIMI) flow grade 0 for a documented or presumed duration of at least 3 months.^7^ The presence of CTO was ascertained from angiography reports. All angiograms were reviewed by a single observer (G.S.). When the report was felt to be inaccurate, the case was submitted for further adjudication by 2 reviewers. (Y.M.S. and R.G.B.C.). In instances of discordance, a fourth adjudicator (A.R.S.) served as the final arbiter.

All procedures in this analysis were performed by a single CTO operator (A.R.S.). Procedural success was defined as successful dilation and stenting of the attempted CTO lesion with the restoration of TIMI-3 flow at the conclusion of the procedure.

All included patients in the study had documented LVEF prior to the PCI and had at least 1 transthoracic echocardiogram after the specified window of follow-up time. These were performed by experienced sonographers and interpreted by level-3 echocardiographers. All methods of reporting LVEF by echocardiography were acceptable for the purpose of this analysis, including qualitative visual assessment, biplane method of disks, or 3D assessment.

### Statistical methods

Categorical variables are expressed as count and percentage and compared using the χ^2^ test, while continuous variables are described as mean ± SD and compared by the *t* test or as medians and IQR and compared using the Mann-Whitney *U* test. Further, we compared the change in LVEF according to procedural success, baseline LVEF, and target CTO vessel in an unadjusted manner. Next, a multivariable logistic regression model was performed to identify the predictors of the binary end point of LVEF improvement of 10% or greater. Variables with *P* value < .2 in the univariable analysis were considered eligible for the multivariable model. The unadjusted and adjusted odds ratios and 95% CIs were reported. A 2-tailed *P* value of .05 was used for significance testing throughout the study. Statistical analysis was conducted using SPSS version 26.0 (IBM Corp).

## Results

We included a total of 142 patients who underwent attempted CTO PCI and had available LVEF at baseline and follow-up. Overall, the mean age was 65.4 ± 10.3 years, 23.9% were women, and 81.0% were White. Comorbidities included diabetes in 51.4%, prior myocardial infarction in 30.3%, heart failure in 35.2%, peripheral arterial disease in 27.5%, chronic kidney disease in 47.2%, and dialysis in 10.6%. Furthermore, 62.7% of the patients had prior PCI, and 21.8% had prior coronary artery bypass grafting.

The attempted native CTO vessels included left anterior descending coronary artery (LAD) in 31.7%, left circumflex artery (LCX) in 17.6%, and right coronary artery (RCA) in 50.0%. The mean multicenter CTO Registry of Japan score (J-CTO score) was 2.0 ± 1.1, including 9.2% of patients with a J-CTO score of 0 (easy), 19.7% with a score of 1 (intermediate), 43.7% with a score of 2 (difficult), and 27.5% with a score of 3 (very difficult). The antegrade approach was used in 95.7% of the cases and procedural success was achieved in 121 patients (85.2%).

Patients who underwent unsuccessful CTO PCI (N = 21) were older (69.7 ± 6.9 vs 64.6 ± 10.6 years; *P* = .008) and had greater body mass index (31.9 ± 6.1 vs 29.1 ± 5.8 kg/m^2^; *P* = .038) and higher baseline LVEF (54.6% ± 10.6% vs 48.2% ± 15.4%; *P* = .024) compared with those who had successful CTO interventions (Table 1). There were no differences in mean J-CTO score or CTO complexity across the groups. Patients with successful procedures were also more likely to be prescribed P2Y_12_ inhibitors, and triple guideline-directed medical therapy at discharge (Table 2).

**Table 1:** Baseline and procedural characteristics of the study population.

	Overall (N = 142)	Successful CTO PCI (n = 121)	Unsuccessful CTO PCI (n = 21)	*P* value
Age, y	65.4 ± 10.3	64.6 ± 10.6	69.7 ± 6.9	.008
Female sex	34 (23.9%)	30 (24.8%)	4 (19.0%)	.569
White race	115 (81.0%)	96 (79.3%)	19 (90.5%)	.230
BMI, kg/m^2^	29.5 ± 5.9	29.1 ± 5.8	31.9 ± 6.1	.038
Hypertension	138 (97.2%)	117 (96.7%)	21 (100.0%)	.398
Diabetes	73 (51.4%)	64 (52.9%)	9 (42.9%)	.396
Dyslipidemia	134 (94.4%)	116 (95.9%)	18 (85.7%)	.062
Prior PCI	89 (62.7%)	73 (60.3%)	16 (76.2%)	.165
Prior CABG	31 (21.8%)	25 (20.7%)	6 (28.6%)	.418
Prior myocardial infarction	43 (30.3%)	39 (32.2%)	4 (19.0%)	.225
Heart failure	50 (35.2%)	45 (37.2%)	5 (23.8%)	.236
Peripheral arterial disease	39 (27.5%)	33 (27.3%)	6 (28.6%)	.902
Cerebrovascular disease	34 (23.9%)	30 (24.8%)	4 (19.0%)	.569
Atrial fibrillation/flutter	41 (28.9%)	35 (28.9%)	6 (28.6%)	.974
Chronic lung disease	15 (10.6%)	12 (9.9%)	3 (14.3%)	.548
Chronic kidney disease	67 (47.2%)	58 (47.9%)	9 (42.9%)	.667
Dialysis	15 (10.6%)	15 (12.4%)	0 (0.0%)	.088
eGFR, mL/min/1.73 m^2^	65.4 ± 25.7	63.8 ± 26.7	74.4 ± 16.7	.082
Serum hemoglobin, g/dL	12.8 ± 2.1	12.6 ± 2.2	13.8 ± 1.5	.004
LVEF, %	49.2 ± 14.9	48.2 ± 15.4	54.6 ± 10.6	.024
Reduced LVEF <50%	53 (37.3%)	50 (41.3%)	3 (14.3%)	.018
Reduced LVEF ≤35%	33 (23.3%)	31 (25.6%)	2 (10.0%)	.107
Native target vessel^a^				
LAD	45 (31.7%)	40 (33.1%)	5 (23.8%)	.400
LCX	25 (17.6%)	24 (19.8%)	1 (4.8%)	.094
RCA	71 (50.0%)	57 (47.1%)	14 (66.7%)	.098
J-CTO score	2.0 ± 1.1	1.9 ± 1.0	2.2 ± 1.3	.321
CTO complexity				.925
Easy (J-CTO score 0)	13 (9.2%)	11 (9.1%)	2 (9.5%)	
Intermediate (J-CTO score 1)	28 (19.7%)	25 (20.7%)	3 (14.3%)	
Difficult (J-CTO score 2)	62 (43.7%)	52 (43.0%)	10 (47.6%)	
Very difficult (J-CTO score ≥3)	39 (27.5%)	33 (27.3%)	6 (28.6%)	
CTO crossing strategy				.169
Antegrade	135 (95.7%)	117 (96.7%)	18 (90.0%)	
Retrograde	6 (4.3%)	4 (3.3%)	2 (10.0%)	
Procedure time, min	107.1 ± 43.6	103.8 ± 45.1	125.9 ± 28.0	.032
Fluoroscopy time, min	40.5 ± 19.6	37.8 ± 19.1	55.7 ± 14.5	<.001
Contrast volume, mL	222.1 ± 91.6	214.0 ± 86.0	269.0 ± 109.8	.011

Values are mean ± SD or n (%).

CABG, coronary artery bypass grafting; CTO, chronic total occlusion; eGFR, estimated glomerular filtration rate; J-CTO score, multicenter CTO Registry of Japan score; LAD, left anterior descending; LCX, left circumflex; LVEF, left ventricular ejection fraction; PCI, percutaneous coronary intervention; RCA, right coronary artery.

a Patients who underwent interventions of the left internal mammary artery (n = 1), or right internal mammary artery (n = 1) were still included in the main analysis but not counted in the analysis by native vessel.

**Table 2:** Rates of medical therapy prescriptions after index CTO PCI procedure.

	Overall (N = 142)	Successful CTO PCI (n = 121)	Unsuccessful CTO PCI (n = 21)	*P* value
Medical therapy at discharge				
Aspirin	123 (86.6%)	104 (86.0%)	19 (90.5%)	.574
P2Y_12_ inhibitor	137 (96.5%)	120 (99.2%)	17 (81.0%)	<.001
DAPT	119 (83.8%)	104 (86.0%)	15 (71.4%)	.095
Statin	132 (93.0%)	111 (91.7%)	21 (100.0%)	.172
High-intensity statin	111 (78.2%)	91 (75.2%)	20 (95.2%)	.004
Nonstatin lipid-lowering therapy	17 (12.0%)	17 (14.0%)	0 (0.0%)	.067
Beta-blockers	111 (78.2%)	93 (76.9%)	18 (85.7%)	.365
ACEI/ARB/ARNI	77 (54.2%)	65 (53.7%)	12 (57.1%)	.771
MRA	13 (9.2%)	12 (9.9%)	1 (4.8%)	.450
SGLT2i	21 (14.8%)	15 (12.4%)	6 (28.6%)	.054
Heart failure GDMT				.024
None	11 (7.7%)	11 (9.1%)	0 (0.0%)	
Single	61 (43.0%)	53 (43.8%)	8 (38.1%)	
Double	50 (35.2%)	39 (32.2%)	11 (52.4%)	
Triple	19 (13.4%)	18 (14.9%)	1 (4.8%)	
Quadruple	1 (0.7%)	0 (0.0%)	1 (4.8%)	
Medical therapy at follow-up^a^				
Beta-blockers	104 (73.2%)	89 (73.6%)	15 (71.4%)	.839
ACEI/ARB/ARNI	69 (48.6%)	62 (51.2%)	7 (33.3%)	.130
MRA	20 (14.1%)	19 (15.7%)	1 (4.8%)	.183
SGLT2i	23 (16.2%)	19 (15.7%)	4 (19.0%)	.701
Heart failure GDMT				.515
None	23 (16.2%)	19 (15.7%)	4 (19.0%)	
Single	53 (37.3%)	43 (35.5%)	10 (47.6%)	
Double	39 (27.5%)	34 (28.1%)	5 (23.8%)	
Triple	23 (16.2%)	22 (18.2%)	1 (4.8%)	
Quadruple	4 (2.8%)	3 (2.5%)	1 (4.8%)	

ACEI, angiotensin-converting enzyme inhibitor; ARB, angiotensin II receptor blocker; ARNI, angiotensin receptor-neprilysin inhibitor; CTO, chronic total occlusion; DAPT, dual antiplatelet therapy; GDMT, guideline-directed medical therapy; MRA, mineralocorticoid receptor antagonist; PCI, percutaneous coronary intervention; SGLT2i, sodium-glucose co–transporter-2 inhibitor.

a At the time of obtaining the follow-up echocardiogram for left ventricular ejection fraction assessment.

After a median time of 8.4 months (IQR, 4.4-12.4) between PCI and the follow-up echocardiogram, patients who had successful CTO PCI experienced an increase in the mean LVEF from 48.2% ± 15.4% at baseline to 51.8% ± 14.2% (Δ LVEF 3.6%; *P* < .001). Notably, 29.8% of those had an improvement in LVEF of 10% or greater. There were no significant changes in LVEF after unsuccessful CTO PCI (54.6% ± 10.6% vs 55.2 ± 11.8%; *P* = .746) (Central Illustration A).

**Central Illustration:**
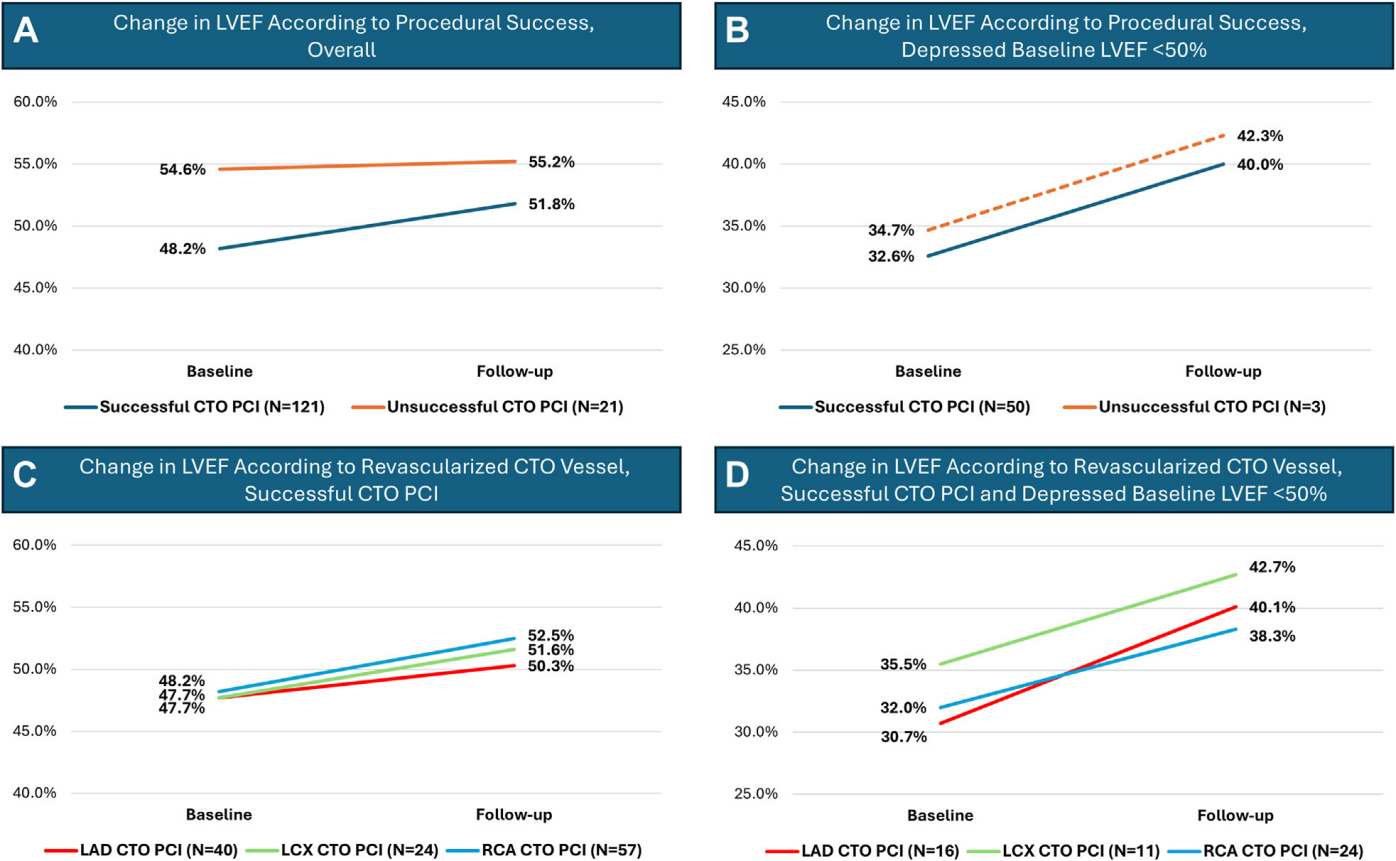
**Change in left ventricular ejection fraction (LVEF) according to different comparison groups.** (**A**) The difference in LVEF at baseline and follow-up according to procedural success in the overall study cohort of 142 patients. (**B**) The difference in LVEF according to procedural success in patients with depressed baseline LVEF less than 50%. Only 3 patients were in the unsuccessful percutaneous coronary intervention (PCI) group with no statistically significant difference from baseline to follow-up (*P* = .222). (**C, D**) The difference in LVEF from baseline to follow-up according to the revascularized chronic total occlusion (CTO) vessel in the overall cohort who underwent successful CTO PCI, as well as those with depressed LVEF who underwent successful CTO PCI, respectively. LAD, left anterior descending; LCX, left circumflex; RCA, right coronary artery.

Among patients with baseline LVEF <50% who underwent successful CTO PCI (N = 50), there was an increase in LVEF from 32.6% ± 9.7% to 40.0% ± 12.9% (Δ LVEF 7.6%; *P* < .001) (Central Illustration B), including 48.0% with ≥10% improvement. There were no significant differences in New York Heart Association functional status at follow-up between successful and unsuccessful PCI. The Canadian Cardiovascular Society class was more favorable at follow-up among patients with successful PCI; however, the difference did not reach statistical significance (Supplemental Table S1).

We compared baseline and procedural characteristics among patients who had LVEF improvement versus those who did not. There were no significant differences in demographics or comorbidities between the 2 groups (Supplemental Table S2). However, patients with ≥10% improvement in LVEF had lower baseline LVEF (38.7% ± 13.4% vs 53.3% ± 13.4%; *P* < .001). Notably, 65.0% of the LVEF improvement group had baseline LVEF <50% and 45.0% had LVEF ≤35% compared with 26.5% and 14.7%, respectively in the group without improvement in LVEF (*P* < .001 for both). There were no significant differences in the target vessels attempted, procedural success rates, or CTO complexity across the groups. Similarly, there were no significant differences in the rates of the prescribed medications on discharge or at follow-up across these groups (Supplemental Table S3).

The change in LVEF in the overall population did not differ according to the attempted CTO vessel after successful PCI. The ΔLVEF was 2.6%, 4.0%, and 4.4% after CTO PCI of LAD, LCX, and RCA, respectively in the overall cohort (Central Illustration C). However, among patients with LVEF <50%, the ΔLVEF was 9.4%, 6.3%, and 7.3% after successful CTO PCI of the LAD, LCX, and RCA, respectively (Central Illustration D).

Multivariable analysis of factors favoring LVEF improvement (10% or greater) after successful CTO indicated that baseline LVEF <50% was a strong independent predictor of LV function improvement at follow-up (adjusted odds ratio, 5.60; 95% CI, 2.27-13.84; *P* < .001). However, the target CTO vessel was not an independent predictor of LVEF improvement ≥10% after adjustment (Table 3).

**Table 3:** Logistic regression analysis for predictors of LVEF improvement ≥10% after successful CTO PCI (N = 121).

	Univariable		Multivariable	
	OR (95% CI)	*P* value	OR (95% CI)	*P* value
Age, per 10-year increase	0.93 (0.70-1.47)	.928	–	–
Female sex	0.65 (0.25-1.69)	.377	–	–
White race	1.44 (0.52-3.97)	.482	–	–
Body mass index	0.97 (0.90-1.04)	.426	–	–
Diabetes	0.72 (0.33-1.58)	.417	–	–
Prior myocardial infarction	0.90 (0.39-2.08)	.797	–	–
Prior PCI	1.24 (0.55-2.77)	.603	–	–
Prior CABG	0.90 (0.34-2.38)	.830	–	–
Peripheral arterial disease	1.52 (0.65-3.57)	.332	–	–
Cerebrovascular disease	1.25 (0.52-3.03)	.621	–	–
Atrial fibrillation/flutter	1.12 (0.48-2.62)	.797	–	–
Baseline serum eGFR	1.02 (1.00-1.03)	.070	1.01 (0.99-1.03)	.370
Baseline serum hemoglobin	1.26 (1.03-1.53)	.024	1.23 (0.98-1.53)	.075
Reduced baseline LVEF <50%	4.54 (1.97-10.43)	<.001	5.60 (2.27-13.84)	<.001
Heart failure GDMT on discharge	1.24 (0.78-1.96)	.358	–	–
Heart failure GDMT at follow-up	1.60 (1.11-2.30)	.012	1.07 (0.70-1.64)	.767
Device therapy at follow-up^a^	1.02 (0.30-3.47)	.972	–	–
CTO LAD	1.02 (0.45-2.33)	.967	–	–
CTO LCX	0.97 (0.36-2.58)	.944	–	–
CTO RCA	1.18 (0.54-2.57)	.678	–	–

Variables with *P* < .2 in the univariable analysis were considered eligible for the multivariable model.

CABG, coronary artery bypass grafting; CTO, chronic total occlusion; eGFR, estimated glomerular filtration rate; GDMT, guideline-directed medical therapy; LAD, left anterior descending; LCX, left circumflex; LVEF, left ventricular ejection fraction OR, odds ratio; PCI, percutaneous coronary intervention; RCA, right coronary artery.

a This included patients with implantable cardioverter defibrillators with or without cardiac resynchronization therapy.

## Discussion

In this single-center experience, we investigated the impact of PCI for CTO on LV function. The main study findings are as follows: (1) after a median follow-up of 8.4 months after successful CTO PCI, there was an overall increase in LVEF by 3.6%, whereas no improvement was observed following unsuccessful interventions; (2) among patients with depressed baseline LVEF <50%, there was a greater degree of LVEF improvement of 7.6%; (3) baseline LVEF <50% was independently associated with a higher likelihood of LV function improvement after CTO PCI; (4) patients who underwent LAD CTO PCI seemed to experience a greater degree of improvement in LVEF compared with those who underwent PCI of the RCA or LCX, specifically in patients with a depressed baseline LVEF. Our hierarchical case review system adds an additional level of rigor to the analysis.

The effect of PCI on LV function in chronically occluded coronary arteries remains a subject of debate. The EXPLORE and REVASC trials both found no significant improvements in LVEF with CTO PCI.^1^^,^^2^ The REVASC trial included patients with stable coronary artery disease and did not show improvement in indexes of regional and global LV function assessed by cardiac magnetic resonance imaging at 6 months. However, in that trial, a significant proportion of patients did not have baseline dysfunction in the CTO segment. Initial LVEF in both groups was 55% to 60%, with the RCA being the predominant CTO vessel (∼ 70%).^2^ Consequently, there was limited potential for improvement, whereas the mean baseline LVEF in our study was 49%. The EXPLORE trial found no overall benefit for LVEF from CTO PCI performed within a week after primary PCI for ST-elevation myocardial infarction, even with baseline LVEF in the 45% range. However, subgroup analysis suggests that early additional PCI may benefit patients with LAD CTO (23% of the cohort).^1^

Observational studies and meta-analyses consistently showed that CTO PCI was associated with improvements in LVEF, being more apparent in patients with depressed LV function. A recent meta-analysis by Megaly et al^8^ showed significant improvements in regional segmental wall thickening and LVEF after CTO PCI, especially in patients with baseline LVEF <45%. In those patients, the LVEF improvement was 5.5% at 6 months after PCI. Other studies reported similar improvement in patients with depressed LV function, ranging from 5% to 10%, following PCI of various vessels.^5^^,^^6^^,^^9^^,^^10^ Our study aligned with previous reports, showing a 3.6% overall improvement in LVEF after a median follow-up of 9.4 months after PCI, which was more significant up to 7.6% among patients with baseline LVEF <50%. This was despite no differences in prescribing guideline-directed medical therapy prior to hospital discharge or at follow-up at the time of LVEF assessment. Further, baseline LVEF <50% was independently associated with greater odds of LV function improvement after successful CTO PCI. On the other hand, patients who had unsuccessful CTO PCI lacked a significant improvement in LV function, but that could be partly related to starting with a higher baseline LVEF compared with the successful PCI group (54.6% vs 48.2%).

Regarding the effect of CTO vessel type on LVEF improvement, a single study found that in patients with ischemic cardiomyopathy (LVEF <40%), LAD CTO PCI led to a significant LVEF improvement of 10.9% at 9 months. This effect was even more noticeable (14% at 6 months) in those with proximal LAD CTO.^11^ In our cohort, LAD CTO PCI in patients with depressed LV function led to an average increase in the absolute value of LVEF by 9.4%, which is consistent with previous findings. However, an independent association with the target vessel could not be established when we looked at the categorical end point of LVEF improvement of at least 10%. This finding may be partly related to the small subgroup size.

Further research is needed to assess the long-term impact of CTO PCI on regional and global LV function. Although other factors, like baseline viability, are under investigation, most patients with CTO lesions and documented ischemia and viability show significant improvements in stress myocardial blood flow and reduced ischemic burden after PCI, though LVEF improvements are minimal.^12^

### Limitations

Our results need to be considered in the light of several limitations. These include the fact that our study was single-center, retrospective, and observational in nature. Further, our study involved a relatively small number of patients, especially patients with unsuccessful CTO PCI, and the subgroup analyses were based on target vessels. This may have limited our ability to detect a statistically significant association with LVEF improvement in the adjusted analysis of patients with reduced LVEF and LAD CTO PCI. Additionally, our analysis lacked a blinded echocardiographic core laboratory. Nevertheless, all echocardiograms were reviewed by experienced level-3 echocardiographers, and our findings were consistent with multiple previous studies in the literature. Moreover, the absence of standardized assessment and reporting of viability may be a limitation in our study, as well as in previously published reports. Notably, potential survivorship bias may have led to an overestimation of the impact of successful CTO PCI on LVEF, as this group may not be fully representative of all patients undergoing the procedure. However, it is important to note that no significant improvement in LVEF was observed in the small subset of patients with unsuccessful PCI. Finally, all our CTO PCI procedures were performed by a single operator using contemporary techniques at a large volume center; hence, the results may not be applicable to other centers or operators with less experience or different procedural techniques. This consistency, however, minimized interoperator variability, which is advantageous in a field heavily dependent on the skillset of operators.

## Conclusion

Successful CTO PCI appears to be associated with a modest improvement in LVEF, particularly in patients who present with reduced baseline LVEF. The degree of LVEF improvement was more significant following successful revascularization of the LAD compared with other coronary vessels, especially in patients with reduced LVEF. Further investigation is warranted to elucidate the mechanisms underlying these differential outcomes and identify patient subgroups who may derive the greatest benefit from CTO PCI.
