# Supplementary material for: Assessment of Left Ventricular Function After Percutaneous Coronary Intervention for Chronic Total Occlusion
Source: J Soc Cardiovasc Angiogr Interv. 2025 Jan 21;4(1):102460. doi: 10.1016/j.jscai.2024.102460 (PMC11887552; doi:10.1016/j.jscai.2024.102460)
Supplement: Supplementary Tables [file mmc1.docx]

**Supplemental Table S1. Comparison of symptoms and functional status before and after CTO PCI:**

|  | **Unsuccessful CTO PCI**  **(N=21)** | **Successful CTO PCI**  **(N=121)** | **P-value** |
| --- | --- | --- | --- |
| Chest pain   - Baseline - Follow-up | 14 (66.7%)  8 (38.1%) | 87 (71.9%)  23 (19.3%) | 0.625  0.056 |
| Dyspnea   - Baseline - Follow-up | 17 (81.0%)  9 (42.9%) | 85 (70.2%)  33 (28.0%) | 0.314  0.171 |
| Fatigue   - Baseline - Follow-up | 8 (38.1%)  6 (28.6%) | 51 (42.5%)  19 (16.0%) | 0.706  0.164 |
| Baseline NYHA   - Class I - Class II - Class III - Class IV | 3 (14.3%)  12 (57.1%)  5 (23.8%)  0 (0.0%) | 23 (19.0%)  59 (48.8%)  38 (31.4%)  1 (0.8%) | 0.150 |
| Follow-up NYHA   - Class I - Class II - Class III - Class IV | 11 (52.4%)  7 (33.3%)  2 (9.5%)  0 (0.0%) | 81 (66.9%)  23 (19.0%)  13 (10.7%)  1 (0.8%) | 0.583 |
| Baseline CCS Class   - Class I - Class II - Class III - Class IV | 4 (19.0%)  11 (52.4%)  6 (28.6%)  0 (0.0%) | 22 (18.3%)  54 (45.0%)  37 (30.8%)  1 (0.8%) | 0.832 |
| Follow-up CCS Class   - Class I - Class II - Class III - Class IV | 10 (47.6%)  7 (33.3%)  2 (9.5%)  0 (0.0%) | 65 (55.1%)  19 (16.1%)  8 (6.8%)  1 (0.8%) | 0.328 |
| CTO: Chronic total occlusion, PCI: Percutaneous coronary intervention, NYHA: New York Heart Association, CCS: Canadian Cardiovascular Society. | | | |

**Supplemental Table S2. Baseline and procedural characteristics according to LVEF improvement ≥10%:**

|  | **LVEF Improvement**  **(N=40)** | **No LVEF Improvement**  **(N=102)** | **P-value** |
| --- | --- | --- | --- |
| Age (years), mean ± SD | 65.3 ± 9.5 | 65.4 ± 10.7 | 0.921 |
| Female sex (%) | 8 (20.0%) | 26 (25.5%) | 0.490 |
| White race (%) | 34 (85.0%) | 81 (79.4%) | 0.445 |
| BMI (kg/m^2^), mean ± SD | 28.8 ± 5.2 | 29.8 ± 6.2 | 0.361 |
| Hypertension (%) | 40 (100.0%) | 98 (96.1%) | 0.204 |
| Diabetes (%) | 20 (50.0%) | 53 (52.0%) | 0.833 |
| Dyslipidemia (%) | 37 (92.5%) | 97 (95.1%) | 0.546 |
| Prior PCI (%) | 27 (67.5%) | 62 (60.8%) | 0.457 |
| Prior CABG (%) | 9 (22.5%) | 22 (21.6%) | 0.904 |
| Prior myocardial infarction (%) | 12 (30.0%) | 31 (30.4%) | 0.964 |
| Heart failure (%) | 19 (47.5%) | 31 (30.4%) | 0.055 |
| Peripheral arterial disease (%) | 12 (30.0%) | 27 (26.5%) | 0.672 |
| Cerebrovascular disease (%) | 11 (27.5%) | 23 (22.5%) | 0.534 |
| Atrial fibrillation / flutter (%) | 12 (30.0%) | 29 (28.4%) | 0.853 |
| Chronic lung disease (%) | 4 (10.0%) | 11 (10.8%) | 0.891 |
| Chronic kidney disease (%) | 16 (40.0%) | 51 (50.0%) | 0.283 |
| Dialysis (%) | 2 (5.0%) | 13 (12.7%) | 0.177 |
| eGFR (mL/min/1.73m^2^), mean ± SD | 70.3 ± 22.5 | 63.5 ± 26.7 | 0.159 |
| Serum hemoglobin (g/dL), mean ± SD | 13.4 ± 2.0 | 12.5 ± 2.1 | 0.023 |
| LVEF (%), mean ± SD | 38.7 ± 13.4 | 53.3 ± 13.4 | <0.001 |
| Reduced LVEF <50% (%) | 26 (65.0%) | 27 (26.5%) | <0.001 |
| Reduced LVEF ≤35% (%) | 18 (45.0%) | 15 (14.7%) | <0.001 |
| CTO vessel   - LAD - LCX - RCA | 13 (32.5%)  8 (20.0%)  19 (47.5%) | 32 (31.4%)  17 (16.7%)  52 (51.0%) | 0.897  0.639  0.709 |
| Procedural success (%) | 36 (90.0%) | 85 (83.3%) | 0.314 |
| J-CTO score, mean ± SD | 1.9 ± 1.0 | 2.0 ± 1.1 | 0.364 |
| CTO complexity (%)   - Easy (J-CTO score 0) - Intermediate (J-CTO score 1) - Difficult (J-CTO score 2) - Very difficult (J-CTO score ≥3) | 4 (10.0%)  9 (22.5%)  18 (45.0%)  9 (22.5%) | 9 (8.8%)  19 (18.6%)  44 (43.1%)  30 (29.4%) | 0.853 |
| CTO crossing strategy (%)   - Antegrade - Retrograde | 38 (95.0%)  2 (5.0%) | 97 (96.0%)  4 (4.0%) | 0.783 |
| Procedure time (min), mean ± SD | 112.6 ± 42.5 | 104.9 ± 44.0 | 0.345 |
| Fluoroscopy time (min), mean ± SD | 40.2 ± 19.2 | 40.6 ± 19.8 | 0.899 |
| Contrast volume (mL), mean ± SD | 224.6 ± 51.7 | 221.1 ± 103.4 | 0.793 |
| LVEF Improvement was defined as an increase of at least 10% at the follow-up echocardiogram compared with baseline.  LVEF: Left ventricular ejection fraction, CTO: Chronic total occlusion, PCI: Percutaneous coronary intervention, CABG: Coronary artery bypass grafting, eGFR: Estimated glomerular filtration rate, LAD: Left anterior descending, LCX: Left circumflex, RCA: Right coronary artery, J-CTO score: Multicenter CTO Registry of Japan score. | | | |

**Supplemental Table S3. Rates of medical therapy prescriptions on discharge after index CTO PCI procedure according to LVEF improvement ≥10%:**

|  | **LVEF Improvement**  **(N=40)** | **No LVEF Improvement**  **(N=102)** | **P-value** |
| --- | --- | --- | --- |
| 1. **Medical Therapy at Discharge** | | | |
| Aspirin (%) | 36 (90.0%) | 87 (85.3%) | 0.459 |
| P2Y_12_ inhibitor (%) | 39 (97.5%) | 98 (96.1%) | 0.679 |
| DAPT (%) | 35 (87.5%) | 84 (82.4%) | 0.454 |
| Statin (%) | 37 (92.5%) | 95 (93.1%) | 0.894 |
| High-intensity statin (%) | 33 (82.5%) | 78 (76.5%) | 0.434 |
| Non-statin lipid lowering therapy (%) | 1 (2.5%) | 16 (15.7%) | 0.029 |
| Beta blockers (%) | 32 (80.0%) | 79 (77.5%) | 0.741 |
| ACEI / ARB / ARNI (%) | 22 (55.0%) | 55 (53.9%) | 0.908 |
| MRA (%) | 5 (12.5%) | 8 (7.8%) | 0.387 |
| SGLT-2i (%) | 8 (20.0%) | 13 (12.7%) | 0.273 |
| Heart failure GDMT (%)   - None - Single - Double - Triple - Quadruple | 2 (5.0%)  16 (40.0%)  15 (37.5%)  7 (17.5%)  0 (0.0%) | 9 (8.8%)  45 (44.1%)  35 (34.3%)  12 (11.8%)  1 (1.0%) | 0.766 |
| 1. **Medical Therapy at Follow-up*** | | | |
| Beta blockers (%) | 33 (82.5%) | 71 (69.6%) | 0.119 |
| ACEI / ARB / ARNI (%) | 24 (60.0%) | 45 (44.1%) | 0.089 |
| MRA (%) | 9 (22.5%) | 11 (10.8%) | 0.071 |
| SGLT-2i (%) | 9 (22.5%) | 14 (13.7%) | 0.202 |
| Heart failure GDMT (%)   - None - Single - Double - Triple - Quadruple | 4 (10.0%)  12 (30.0%)  12 (30.0%)  9 (22.5%)  3 (7.5%) | 19 (18.6%)  41 (40.2%)  27 (26.5%)  14 (13.7%)  1 (1.0%) | 0.093 |
| *At the time of obtaining the follow-up echocardiogram for LVEF assessment.  CTO: Chronic total occlusion, PCI: Percutaneous coronary intervention, DAPT: Dual antiplatelet therapy, ACEI: Angiotensin-converting enzyme inhibitor, ARB: Angiotensin II receptor blocker, ARNI: Angiotensin receptor-neprilysin inhibitor, MRA: Mineralocorticoid receptor antagonist, SGLT-2i: Sodium-glucose co-transporter-2 inhibitor, GDMT: Guideline-directed medical therapy. | | | |
